# Protein tyrosine kinase 6 promotes ERBB2-induced mammary gland tumorigenesis in the mouse

**DOI:** 10.1038/cddis.2015.210

**Published:** 2015-08-06

**Authors:** M Peng, S M Ball-Kell, A L Tyner

**Affiliations:** 1 Department of Biochemistry and Molecular Genetics, University of Illinois at Chicago, Chicago, IL, USA

## Abstract

Protein tyrosine kinase 6 (PTK6) expression, activation, and amplification of the *PTK6* gene have been reported in ERBB2/HER2-positive mammary gland cancers. To explore contributions of PTK6 to mammary gland tumorigenesis promoted by activated ERBB2, we crossed *Ptk6^−/−^* mice with the mouse mammary tumor virus-ERBB2 transgenic mouse line expressing activated ERBB2 and characterized tumor development and progression. ERBB2-induced tumorigenesis was significantly delayed and diminished in mice lacking PTK6. PTK6 expression was induced in the mammary glands of ERBB2 transgenic mice before tumor development and correlated with activation of signal transducer and activator of transcription 3 (STAT3) and increased proliferation. Disruption of PTK6 impaired STAT3 activation and proliferation. Phosphorylation of the PTK6 substrates focal adhesion kinase (FAK) and breast cancer anti-estrogen resistance 1 (BCAR1; p130CAS) was decreased in *Ptk6*^−/−^ mammary gland tumors. Reduced numbers of metastases were detected in the lungs of *Ptk6^−/−^* mice expressing activated ERBB2, compared with wild-type ERBB2 transgenic mice. PTK6 activation was detected at the edges of ERBB2-positive tumors. These data support roles for PTK6 in both ERBB2-induced mammary gland tumor initiation and metastasis, and identify STAT3, FAK, and BCAR1 as physiologically relevant PTK6 substrates in breast cancer. Including PTK6 inhibitors as part of a treatment regimen could have distinct benefits in ERBB2/HER2-positive breast cancers.

Breast cancer remains the second leading cause of death for women in the United States.^1^ The 20–30% incidence of overexpression of the epidermal growth factor receptor family tyrosine kinase ERBB2 (HER2, Neu) in breast cancer^2, 3^ has made it a prominent therapeutic target.^4^ ERBB2 signaling depends on its heterodimerization with another ERBB family member, often ERBB3 or ERBB1 (epidermal growth factor receptor), which leads to activation of the phosphoinositide 3-kinase-AKT pathway. The ERBB2 monoclonal antibody trastuzumab (Herceptin) is an established therapeutic option, but *de novo* and acquired therapeutic resistance to trastuzumab is an important clinical problem.^5^ Variations among signaling proteins present downstream of ERBB2 can contribute to the development of resistance to trastuzumab and other drugs targeting the ERBB2 pathway.

The intracellular tyrosine kinase PTK6 (also called BRK for Breast tumor kinase) is an intracellular tyrosine kinase that is evolutionarily related to SRC family kinases.^6^ PTK6 is overexpressed in a majority of human breast cancers and in most breast tumor cell lines.^7, 8, 9, 10^ Previously, we detected PTK6 expression in a normal mammary gland human breast tissue array, as well as in a human breast tumor array using immunohistochemistry. Phosphorylation of tyrosine residue 342 in the PTK6 activation loop promotes its activation. Interestingly, active PTK6 was only detected in human breast tumors, suggesting that PTK6 may have kinase-independent functions in normal human breast tissue that are distinct from its cancer-promoting activities at the membrane.^9^

A significant correlation between expression of PTK6 and ERBB2 (HER2) has been reported in breast tumors. Several studies have indicated that PTK6 and ERBB2 are coexpressed in human breast tumors and PTK6 promotes ERBB2 oncogenic signaling in human breast tumor cell lines.^11, 12, 13, 14^ PTK6 was not detected in the normal mouse mammary gland, but we found that it was induced and activated in mouse mammary gland tumors, including those that developed in mouse mammary tumor virus (MMTV)-ERBB2 transgenic mice.^15^ Previously identified substrates of PTK6 that have been shown to participate in ERBB2 oncogenic signaling include the transcription factor signal transducer and activator of transcription 3 (STAT3),^15, 16^ focal adhesion kinase (FAK),^17^ and breast cancer anti-estrogen resistance 1 (BCAR1 also known as p130CAS).^18^

Our group previously generated a *Ptk6* null mouse model and characterized its phenotype in the gastrointestinal tract^19, 20, 21^ and skin,^22^ tissues where PTK6 is expressed in differentiated epithelial cells. Disruption of *Ptk6* led to increased growth and impaired differentiation in the small intestine.^21^ However, when *Ptk6* was induced in small intestinal crypt progenitor cells following DNA damage, loss of *Ptk6* impaired DNA-damage-induced apoptosis.^20^ Interestingly, although PTK6 has roles in promoting differentiation and apoptosis in normal epithelia, it can also promote tumorigenesis in both the colon and skin. Disruption of *Ptk6* led to resistance to azoxymethane/dextran sodium sulfate-induced tumorigenesis in colon^19^ and impaired UVB-induced tumorigenesis in the skin.^22^ These data demonstrate that PTK6 has context-specific functions that differ depending on tissue and cell type.

Here we explored the impact that disrupting *Ptk6* has on mammary gland tumorigenesis in the MMTV-ERBB2 mouse model. We found that PTK6 promotes ERBB2-induced tumorigenesis and disruption of *Ptk6 *in vivo** leads to inhibition of STAT3 in pre-tumorigenic mammary glands, and decreased FAK and BCAR1 phosphorylation and activation in mammary gland tumors. Our findings indicate that the induction of PTK6 in ERBB2-induced tumors is physiologically significant, and suggest that targeting PTK6 in ERBB2-positive breast tumors could restrain several signaling pathways that have critical roles in ERBB2-induced tumorigenesis.

## Results

### Disruption of *Ptk6* impairs ERBB2-induced mammary gland tumorigenesis

Previously, we reported expression and activation of PTK6 in mammary gland tumors that developed in MMTV-ERBB2 transgenic mice.^15^ To determine contributions of PTK6 to ERBB2 tumorigenesis, we crossed MMTV-ERBB2 mice (B2) with *Ptk6^−/−^* mice that were backcrossed greater than eight generations into the FVB/NJ strain. No phenotype was detected in the mammary glands of FVB/NJ *Ptk6^−/−^* mice; morphology of ducts and alveoli appeared normal and no lactation defects were observed. Three lines of virgin female MMTV-ERBB2 transgenic mice with different *Ptk6* genotypes were maintained (B2;*Ptk6*^+/+^, B2;*Ptk6*^+/−^, and B2;*Ptk6^−/−^*). Palpable mammary gland tumors could be detected in some B2;*Ptk6*^+/+^ animals by 180 days, and by day 210, all of the B2;*Ptk6*^+/+^ animals had developed one or multiple tumors (*n*=17). B2;*Ptk6*^+/−^ mice developed mammary gland tumors later than B2;*Ptk6*^+/+^ animals, and with a lower occurrence and by day 210, ~25% of the B2;*Ptk6*^+/−^ animals remained tumor free. However, all B2;*Ptk6*^+/−^ mice developed palpable tumors by day 240 (*n*=16).

Complete disruption of *Ptk6* markedly delayed tumor initiation. In contrast to B2;*Ptk6*^+/+^ and B2;*Ptk6*^+/−^ animals, no tumors were detected in any of the B2;*Ptk6^−/−^* animals by day 210 (*n*=20). 92% of B2;*Ptk6^−/−^* animals remained tumor free up to 240 days, in contrast to all of the *Ptk6*-expressing mice that had developed mammary gland tumors. By 260 days, 58% of the B2;*Ptk6^−/−^* animals remained tumor free, whereas all B2;*Ptk6*^+/+^ mice had reached humane end points and mice were killed. The average time required for detection of palpable mammary gland tumors was 184, 201, and 306 days for *Ptk6*^+/+^, *Ptk6*^+/−^, and *Ptk6^−/−^*, respectively. Some B2;*Ptk6^−/−^* animals remained tumor free for 1 year. Although most B2;*Ptk6*^−/−^ mice developed breast tumors after 12 months, tumor initiation was significantly delayed and the survival time was increased. B2;*Ptk6*^+/+^ animals were maintained until they reached the humane end point (tumor >2 cm). B2;*Ptk6^−/−^* animals were killed at later time points when tumor burden (mass) was similar to the B2;*Ptk*6^+/+^ mice for analyses of tumors and tumor metastasis.

**Figure 1:**
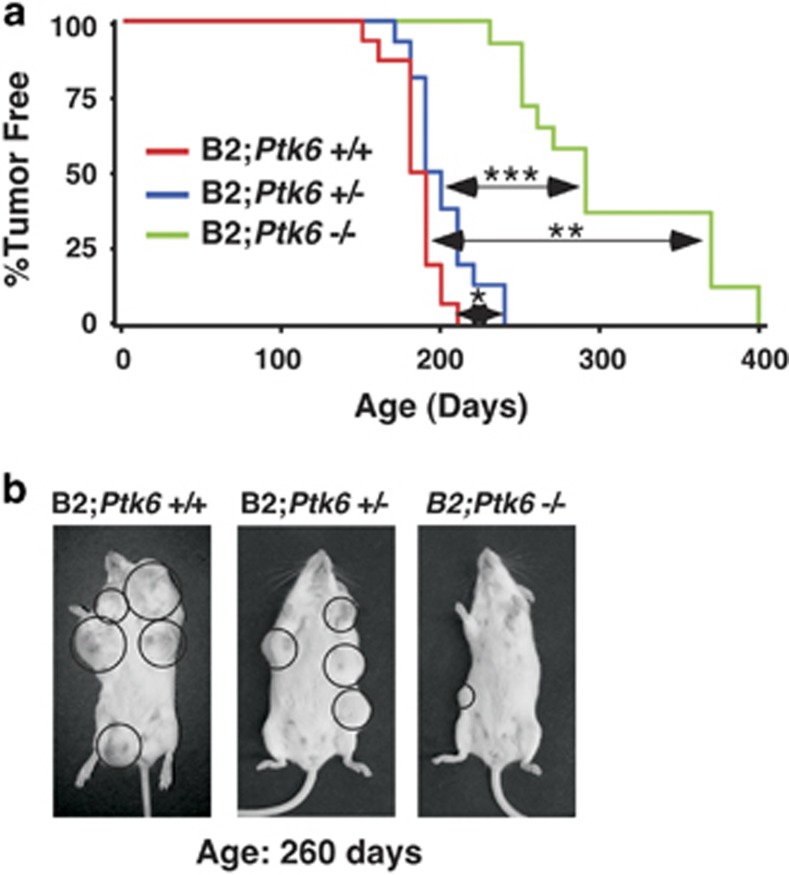
Loss of PTK6 expression delays MMTV-ERBB2-induced mammary gland tumor formation in mice. (**a**) Female MMTV-ERBB2 mice with different *Ptk6* genotypes (B2;*Ptk6*^+/+^; B2;*Ptk6*^+/−^; B2;*Ptk6^−/−^*) displayed distinct mammary gland tumor formation kinetics. *P*-values: *P=0.01 and **, ***P<0.001 between groups indicated by arrows; *n*=17 (B2;*Ptk6*^+/+^); *n*= 16 (B2;*Ptk6*^+/−^); *n*= 20 (B2;*Ptk6*^−/−^). (**b**) Three littermates with different *Ptk6* genotypes displayed different tumor burdens at 260 days of age because of differences in the timing of tumor initiation. Mammary gland tumors are circled

Kinetics of mammary tumor onset in *Ptk6*^+/+^, *Ptk6*^+/−^, and *Ptk6^−/−^*/MMTV-ERBB2 virgin female mice is shown in Figure 1a. Tumor growth in three representative MMTV-ERBB2 littermates with different *Ptk6* genotypes at 260 days of age is shown in Figure 1b. These data demonstrate that systemic disruption of *Ptk*6 results in a dramatic reduction in ERBB2-induced tumorigenesis *in vivo*.

### PTK6 promotes proliferation and STAT3 activation in MMTV-ERBB2 transgenic mammary glands before tumor development

**Figure 2:**
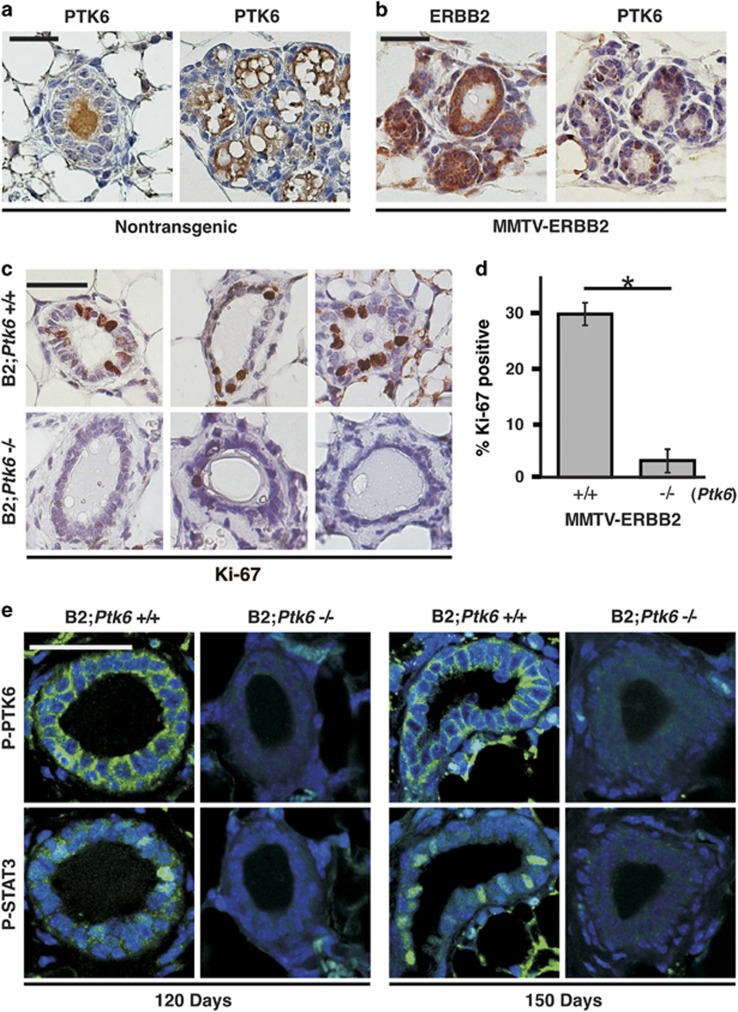
PTK6 expression is induced in pre-tumorigenic MMTV-ERBB2 mammary glands and promotes STAT3 activation and proliferation. (**a**) PTK6 was not expressed in wild-type mouse mammary glands in the mature animals, regardless of the developmental states of the glands (left panel, normal virgin mammary gland; right panel, lactating mammary gland). The brown signal in the center of the glands does not include the epithelial cells and represents background. (**b**) PTK6 expression is induced by ectopic ERBB2. ERBB2 and PTK6 are coexpressed in epithelial cells of a 5-month-old MMTV-ERBB2 PTK6 transgenic mouse. (**c**) Proliferation is impaired in MMTV-ERBB2-positive mammary glands following disruption of *Ptk6*. Proliferation in pre-tumorigenic mammary gland epithelial cells was examined by Ki-67 staining. Each panel represents a different animal. (**d**) Proliferation in mammary gland epithelial cells was quantified by normalizing Ki-67-positive epithelial cells to the total number of epithelial cells. Each group contains five animals, and five different random views were counted per animal. The difference in percent of Ki67-positive cells in B2;*Ptk6*^+/+^ (28.5%) and B2;*Ptk6^−/−^* (2.8%) is statistically significant (*P<0.001). (**e**) Active PTK6 (P-Y342) and active phospho-STAT3 (PY705) can be detected in the same areas in the B2;*Ptk6*^+/+^ mammary glands. In contrast, active phospho-STAT3 was not detectable in B2;*Ptk6^−/−^*animals. The size bars represent 50 *μ*m

PTK6 protein is not detectable in normal mammary gland epithelial cells of nontransgenic mice throughout development (Figure 2a). However, strong PTK6 expression was detected in ERBB2-positive mammary glands (Figure 2b), which corresponded with increased proliferation measured by examining the Ki-67-labeling index using immunohistochemistry (Figures 2c and d). These data indicate that PTK6 expression is induced downstream of ERBB2, and that PTK6 promotes ERBB2-regulated cell proliferation in the mammary gland.

PTK6 activity was measured in MMTV-ERBB2 transgenic mouse mammary glands by examining phosphorylation of tyrosine residue 342 (P-Y342) in the PTK6 activation loop, which serves as a marker for its activation.^23^ Data from two different pairs of mice at age 120 days and 150 days are shown in Figure 2e. Active PTK6 was localized at the membrane in mammary gland epithelial cells, and was only detected in wild-type MMTV-ERBB2 mice demonstrating the specificity of the antibody for PTK6. STAT3 activation requires phosphorylation at tyrosine residue 705 in its carboxyl terminus, and this residue has been identified as a substrate for PTK6.^16^ Immunofluorescence staining of serial sections revealed increased levels of active phospho-STAT3 in mammary gland epithelial cells with increased expression and activation of PTK6 (Figure 2e).

**Figure 3:**
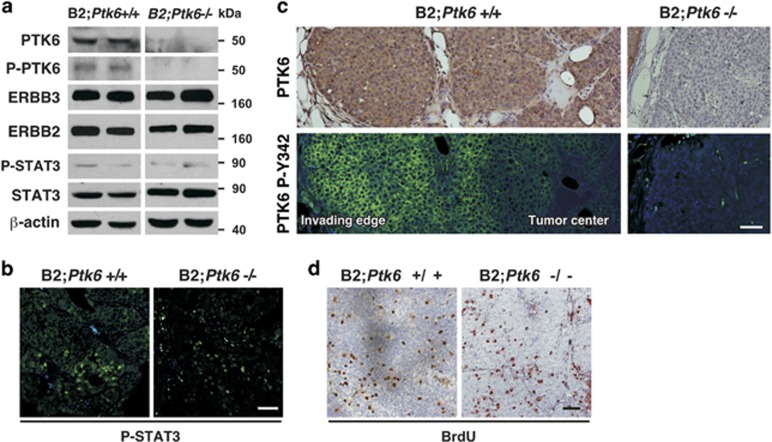
PTK6 is induced throughout ERBB2-induced tumors, but it is activated primarily at the invading edges. (**a**) Protein lysates from ERBB2 transgenic mammary gland tumors were subjected to immunoblotting. Expression of total and active PTK6 was detected only in B2;*Ptk6*^+/+^ lysates. Active P-STAT3 levels were not significantly different between B2;*Ptk6*^+/+^ and B2;*Ptk6^−/−^* animals. (**b**) Cells with active P-STAT3 were detected in B2;*Ptk6*^+/+^ and B2;*Ptk6^−/−^* tumors. Although heterogeneous staining could be detected within tumors, a significant difference in the number of P-STAT3-positive cells was not observed. (**c**) Total PTK6 is ubiquitously expressed in ERBB2 mammary gland tumors as shown by immunohistochemistry, but active PTK6 was primarily localized to the invading edges of the tumors but not in the center. Panels on the right are controls that demonstrate mammary gland tumors that developed in B2;*Ptk6*^−/− ^mice are negative for total and active PTK6. (**d**) BrdU incorporation in breast tumors from B2;*Ptk6*^+/+^ and B2;*Ptk6^−/−^* animals with similar tumor burden was examined by immunohistochemistry. The size bars represent 50 *μ*m

In contrast to the pre-tumorigenic mammary gland, STAT3 activation and proliferation were not reproducibly higher in well-established *Ptk6*^+/+^ MMTV-ERBB2 tumors (Figures 3a and b). STAT3 activation was examined by immunoblotting (Figure 3a) and immunohistochemistry (Figure 3b). Comparable levels of P-STAT3 were detected by immunoblotting in both genotypes, and P-STAT3-positive nuclei could be detected in *Ptk6*^+/+^ and *Ptk6*^−/−^ tumors. Active P-PTK6 was detected in *Ptk6*^+/+^ but not in *Ptk6^−/−^* tumors, as expected (Figures 3a and c). Although total PTK6 protein levels were distributed throughout the mammary gland tumors, activation of PTK6 was detected most strongly at the edges of the tumors (Figure 3c). BrdU incorporation into breast tumors was not significantly different and tumor size did not vary significantly between *Ptk6*^+/+^ and *Ptk6*^−/−^ mice, from the time of the initial tumor palpation until the humane end point (Figure 3d). These data suggest that the PTK6 may be more important for mammary gland tumor initiation rather than growth, or its effect on proliferation could be masked by other strong driver oncogenes like ERBB2 itself.

### PTK6 regulates tyrosine phosphorylation of FAK and BCAR1 and metastasis of mammary gland tumors to the lung

**Figure 4:**
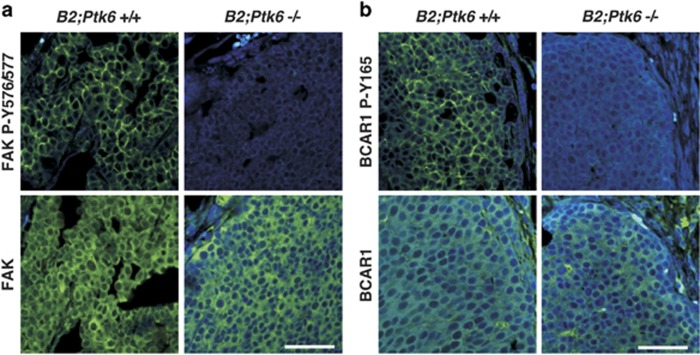
Disruption of *Ptk6* impairs tyrosine phosphorylation of FAK and BCAR1 in ERBB2-positive mammary gland tumors. (**a**) Activated FAK (P-Y576/577) and total FAK expression were examined in MMTV-ERBB2-positive mammary gland tumors. Disruption of *Ptk6* gene led to dramatically reduced levels of activated FAK phosphorylated at tyrosine residues 576/577. (**b**) Phosphorylated BCAR1 (P-Y165) and total BCAR1 expression were examined in MMTV-ERBB2-positive mammary gland tumors. Disruption of *Ptk6* led to reduced phosphorylation of BCAR1. Both P-FAK and P-BCAR1 were primarily localized to the invading edges of the tumors, matching the expression pattern of P-PTK6 (Figure 3c). The size bars represent 50 *μ*m

Recent studies identified FAK^17^ and BCAR1^18^ as direct PTK6 substrates. Both FAK and BCAR1 have been shown to have important roles in ERBB2-regulated tumorigenesis and metastasis in mouse models. Therefore, we examined FAK and BCAR1 expression and tyrosine phosphorylation in *Ptk6*^+/+^ and *Ptk6^−/−^* MMTV-ERBB2 transgenic mice. Comparable expression of FAK was detected in mammary gland tumors that formed in *Ptk6*^+/+^ and *Ptk6^−/−^* MMTV-ERBB2 transgenic mice, but activating tyrosine phosphorylation of FAK at tyrosine residues 576/577 was impaired in *Ptk6^−/−^* tumors (Figure 4a). Similarly, BCAR1 expression was detected in both genotypes, but phosphorylation of the BCAR1 substrate domain at tyrosine residue 165 was impaired in *Ptk6*^−/−^ tumors (Figure 4b). Interestingly, both phospho-FAK and phospho-BCAR1 were concentrated near the edge of the tumors, corresponding with active P-PTK6 localization (Figure 3c). These data indicate that PTK6 has an important functional role in regulating both FAK and BCAR phosphorylation in mammary gland tumors *in vivo*.

**Figure 5:**
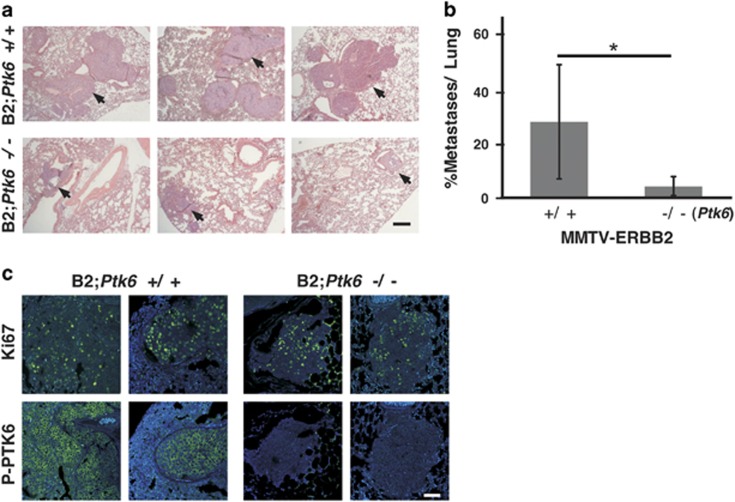
Disruption of *Ptk6* impairs mammary gland tumor metastasis to the lung. (**a**) Lung sections from six B2;*Ptk6*^+/+^ and six B2;*Ptk6^−/−^* animals with similar tumor burden and similar-sized primary tumors were stained with hematoxylin and eosin, and data from three different representative pairs are shown. Fewer intravascular (tumor emboli) and parenchymal masses (arrows) were detected in B2;*Ptk6*^−/−^ lungs. Representative sections are shown for each animal. The size bar represents 200 *μ*m. (**b**) Contributions of metastases to total lung tissue were quantified. B2;*Ptk6*^+/+^: *n*=9, B2*;Ptk6*^−/−^: *n*=6. *P-value=0.013. (**c**) Activating phosphorylation of PTK6 is detected in the metastatic tumor nodules/emboli in the lungs, although proliferation as measured by Ki-67 staining was not statistically different between B2;*Ptk6*^+/+^ and B2;*Ptk6*^−/−^ mice. Sections from two different animals from each group are shown. The size bar represents 50 *μ*m

To examine tumor metastasis, we collected lung tissues from *Ptk6*^+/+^ and *Ptk6*^−/−^ MMTV-ERBB2 transgenic mice with similar total tumor burden, which included mice aged 243±15 days (*Ptk6*^+/+^) and 364±33 days (*Ptk6^−/−^*). Multiple intravascular (tumor emboli) and parenchymal (primarily mammary gland carcinoma, solid type) masses were quantified and normalized to the area of normal lung tissue in tissue sections (Figure 5a). The percent metastases (area of lung occupied by neoplastic mammary gland epithelial cells) was significantly lower in *Ptk6*^−/−^ lungs than in *Ptk6^+/+^* lungs (Figure 5b). Active PTK6 was detected in lung tumor nodules, but a significant decrease in Ki67 was not detected in *Ptk6^−/−^* lung metastases, suggesting that although PTK6 promotes primary tumor metastasis, its activation does not contribute to proliferation in metastatic lesions.

## Discussion

ERBB2 is overexpressed in ~30% of human breast tumors and this correlates with a worse prognosis and clinical outcome.^3, 24^ PTK6 is expressed and activated^9^ in human breast tumors that overexpress ERBB2. We show that PTK6 contributes to both tumor initiation and metastasis in the MMTV-ERBB2 mouse model of breast cancer. We previously showed that PTK6 expression is induced in MMTV-ERBB2 transgenic mouse mammary gland tumors,^15^ and here we demonstrate that PTK6 expression can be detected in the mammary glands of these mice before tumor development. A recent study found that ERBB2 can promote PTK6 protein stability through negative regulation of calpain, a calcium-dependent, non-lysosomal cysteine protease, in breast cancer cell lines.^25^ PTK6 expression is also regulated at the transcriptional^26^ and post transcriptional^27^ levels by HIF-1*α*. HIF-1 expression is positively regulated by ERBB2,^28^ and it is required for ERBB2-mediated mammary gland tumor growth.^29^

PTK6 promotes activating phosphorylation of STAT3 at tyrosine residue 705.^16^ In a previous study, STAT3 activation was detected in MMTV-PTK6 transgenic mouse mammary glands but not in control nontransgenic mammary glands, supporting a role for PTK6 in promoting STAT3 activation.^15^ Here, our data suggest that the initial induction of PTK6 in the ERBB2-positive mouse mammary gland has a distinct role in promoting STAT3 activation and epithelial cell proliferation in the pre-tumorigenic mammary gland (Figure 2). Before mammary gland tumor formation, activating phosphorylation of STAT3 was detected in mammary glands of MMTV-ERBB2 transgenic mice expressing PTK6, but not in ERBB2-positive *Ptk6*^−/−^ mammary glands, and this correlated with higher numbers of Ki-67-positive cells (Figure 2). However, we could not detect reproducible quantifiable differences in STAT3 activation or mammary gland epithelial cell proliferation in established *Ptk6*^+/+^ and *Ptk6^−/−^* ERBB2-positive tumors (Figure 3).

STAT3 has been shown to promote tumor initiation of different tumor types, including those of the gastrointestinal tract and skin (reviewed in refs ^30, 31,^ ^32^), and PTK6 was previously shown to promote STAT3 activation and tumorigenesis in mouse models of colon and skin cancer.^19^^,^^22^ We show that mammary tumor latency is increased in *Ptk6^−/−^* MMTV-ERBB2 mice (Figure 1), and our studies suggest that PTK6-mediated regulation of STAT3 activation promotes ERBB2-induced mammary gland tumor initiation. STAT3 has been shown to contribute to mammary gland tumor progression and metastasis in established mouse models,^33, 34^ and activation of STAT3 has been detected in human breast cancer stem cell models.^35^

We found that disruption of *Ptk6* resulted in reduced metastasis of similar-sized primary tumors to the lungs when comparing *Ptk6*^+/+^ and *Ptk6*^−/−^ MMTV-ERBB2 transgenic mice (Figure 5). PTK6 can have an impact on tumor progression and metastasis through its regulation of FAK and BCAR1. FAK is an intracellular tyrosine kinase that is overexpressed and/or activated in several types of cancers with established roles in regulating tumor progression and metastasis.^36^ We determined that *Ptk6*^+/+^ MMTV-ERBB2 mice exhibited increased levels of active FAK, phosphorylated on tyrosine residues 576/577 (Figure 4a). Disruption of the gene-encoding FAK in the mouse mammary gland blocked mammary gland tumor progression,^37^ but did not appear to be required for tumor induction^38^ in ERBB2 transgenic mice. Conditional disruption of FAK in MMTV-PyMT transgenic mice led to delayed and reduced tumor formation and suppression of tumor progression.^39^

We detected impaired phosphorylation of BCAR1 at tyrosine 165 in its substrate domain in ERBB2-induced mammary gland tumors with disruption of *Ptk6* (Figure 4b). BCAR1 is a scaffold protein that promotes protein–protein interactions and regulates aspects of cell migration, proliferation, and apoptosis. It has an essential role during early development, and has also been implicated in promoting tumorigenesis. BCAR1 was identified in a screen for genes that confer breast tumor resistance to anti-estrogens.^40^ Transgenic expression of BCAR1 in the MMTV-ERBB2 mouse model led to shorter latency of tumor formation compared with expression of ERRB2 alone.^41^ Knockdown of BCAR1 impaired cell migration and invasion of cells expressing active ERBB2.^42^ Phosphorylation of tyrosine residues in the BCAR substrate domain provides docking sites for signaling proteins and has been implicated in promoting migration and cell survival.^43, 44^ We previously determined that tyrosine residues in the BCAR1 substrate domain can be phosphorylated by PTK6, and that BCAR1 was important for oncogenic signaling in prostate cancer cells.^18^

Several studies with human tumor cell lines have suggested PTK6 contributes to ERBB2-induced breast cancer. A recent study showed that siRNA mediated knockdown of both PTK6 and ERBB2 in human breast cancer cell lines additively impairs xenograft tumor growth.^45^ Recently, PTK6 was identified as a kinase that is differentially regulated in ERBB2-positive breast cancer cells that develop resistance to lapatinib,^46^ and shRNA mediated knockdown of PTK6 reduced growth and promoted apoptosis of lapatinib-resistant ERBB2-positive breast cancer cell lines.^47^ For the first time, we have established that endogenous wild-type PTK6 signaling contributes to ERBB2-induced mammary gland tumorigenesis and metastasis *in vivo* in a mouse model of ERBB2-induced breast cancer. We have determined that PTK6 promotes tumor initiation and progression and is important for regulation of STAT3, FAK, and BCAR1. Disruption of *Ptk6* significantly delayed and reduced ERBB2-induced mammary gland tumor formation and metastasis, providing strong rationale for therapeutically targeting PTK6 alone or in combination with other agents in ERBB2/HER2-positive breast cancers.

## Materials and methods

### Mice

*Ptk6* null mice (B6-*Ptk6*^tm1Aty^) in the C57BL/6 strain^19^ were backcrossed with the FVB/N inbred strain (Harlan Laboratories, Frederick, MD, USA) for at least eight generations to generate *Ptk6* null mice in FVB/N background. FVB-*Ptk6*^tm1Aty^ were then crossed with FVB-MMTV-ERBB2 transgenic mice^48^ (Jackson Laboratories, Bar Harbor, ME, USA, Stock number 005038, FVB-Tg(MMTV-Erbb2)^NK1Mul/J^) to produce ERBB2;*Ptk6^−/−^* animals. These mice carry activated rat c-Neu (*Erbb2*) (Val^664^ to Glu^664^) under the control of the MMTV long-terminal repeat (LTR).

Tissues from age-matched female mice were used in all experiments, and FVB/NJ nontransgenic mice were used for maintaining the lines and nontransgenic controls. Animals were palpated one or two times a week for subcutaneous mass from the age of 8 weeks.

### Tissue preparation and analysis

Mammary gland tissues were harvested from sexually mature female animals aged from 4 to 6 months at the estrus/metestrus phase, to control for hormonally regulated changes in proliferation.^49^ The phase of the estrus cycle was determined by vaginal smear.^50^ Mammary gland tumors were harvested when the animals reached the criteria for humane end points. Tissues were fixed in 10% buffered formalin (Fisher Scientific, Pittsburgh, PA, USA) for 24 h and then transferred to 70% ethanol before processing. Paraffin-embedded tissues were sectioned at 5 *μ*m and stained with antibodies and then counterstained with hematoxylin. Mammary gland tumor sections were evaluated by a veterinary pathologist (S M Ball-Kell).

For metastatic lung tumors, lungs from at least six animals per genotype were harvested and embedded in paraffin. Lungs were then serially sectioned at 500 *μ*m intervals and three slides from each animal were stained with hematoxylin and eosin for histopathologic evaluation. The metastatic nodules present in five photographic images were randomly selected from each slide and quantified. The relative sizes of the lung metastases were measured by Image J^51^ and normalized to the area of normal lung tissue. Owing to the uneven distribution of metastatic tumors in the lung, the slides for each animal with the highest number of metastatic lung nodules/normal lung tissue ratio were compared.

### Analysis of protein expression

Preparation of protein lysates and immunoblotting, immunohistochemistry, and immunofluorescence staining were performed as previously described.^15^ Anti-mouse PTK6 (C-17), anti-ERBB2, anti-ERBB3, and anti-FAK antibodies were purchased from Santa Cruz Biotechnology (Santa Cruz, CA, USA). Antiphospho-PTK6 Tyr-342 (P-Y342) antibody was purchased from Millipore (Bedford, MA, USA). Total STAT3 and phospho-STAT3 PY705, phospho-FAK P-Y576/577, and phospho-BCAR1 P-Y165 antibodies were purchased from Cell Signaling Technology (Danvers, MA, USA). Anti-BCAR1 antibody was obtained from BD Bioscience (San Jose, CA, USA), and anti-Ki-67 antibody from Abcam (Cambridge, MA, USA). Anti *β*-actin (AC-15) was purchased from Sigma-Aldrich (St Louis, MO, USA). Sheep anti-mouse and donkey anti-rabbit antibodies were purchased from GE Healthcare Biosciences (Pittsburgh, PA, USA).

### Statistics

For Figure 1a, survival analysis was used to estimate and compare the distributions of time to tumor formation. Three groups of mice with the genotypes of B2;*Ptk6*^+/+^, B2;*Ptk6*^+/−^, and B2;*Ptk6^−/−^* were plotted for Kaplan–Meier survival curves. Statistical analysis was conducted using the survival analysis package PROC LIFETEST in the software SAS 9.3 (SAS, Cary, NC, USA). *P*-values from log-rank tests for comparing survival curves were generated and a difference was considered statistically significant if the *P*-value was equal to or less than 0.05. For Figures 2d and 5b, quantitative data are shown as the mean±S.D. *P*-values were determined using the two-tailed Student's *t-*test (Microsoft Excel, 2010).

## Abbreviations

PTK6: Protein tyrosine kinase 6
BRK: Breast tumor kinase
MMTV: mouse mammary tumor virus
STAT3: signal transducer and activator of transcription 3
FAK: focal adhesion kinase
BCAR1: breast cancer anti-estrogen resistance 1
B2;*Ptk6*^+/+^: MMTV-ERBB2 transgenic mouse with wild-type *Ptk6*
B2;*Ptk6^−/−^*: *Ptk6^/−^* MMTV-ERBB2 transgenic mouse
